# Incidence of hyperthyroidism in patients with bipolar or schizoaffective disorder with or without lithium: 21-year follow-up from the LiSIE retrospective cohort study

**DOI:** 10.1177/20451253231151514

**Published:** 2023-02-09

**Authors:** Ingrid Lieber, Michael Ott, Robert Lundqvist, Mats Eliasson, Ursula Werneke

**Affiliations:** Department of Psychiatry, Sunderby Hospital, 97180 Luleå, Sweden; Sunderby Research Unit, Division of Psychiatry, Department of Clinical Sciences, Umeå University, Umeå, Sweden; Department of Public Health and Clinical Medicine, Medicine, Umeå University, Umeå, Sweden; Sunderby Research Unit, Department of Public Health and Clinical Medicine, Medicine, Umeå University, Umeå, Sweden; Sunderby Research Unit, Department of Public Health and Clinical Medicine, Medicine, Umeå University, Umeå, Sweden; Sunderby Research Unit, Division of Psychiatry, Department of Clinical Sciences, Umeå University, Umeå, Sweden

**Keywords:** bipolar disorder, hyperthyroidism, incidence rate, lithium, schizoaffective disorder, thyroiditis, thyrotoxicosis

## Abstract

**Background:**

Lithium-associated hyperthyroidism is much rarer than lithium-associated hypothyroidism. Yet, it may be of substantial clinical significance for affected individuals. For instance, lithium-associated hyperthyroidism could destabilise mood, mimic manic episodes and impact physical health. Only few studies have explored incidence rates of lithium-associated hyperthyroidism. Even fewer studies have compared incidence rates according to lithium exposure history.

**Objectives:**

To determine the impact of lithium treatment on the incidence rate of hyperthyroidism in patients with bipolar or schizoaffective disorder and assess its aetiology.

**Design:**

This study is part of the LiSIE (Lithium – Study into Effects and Side Effects) retrospective cohort study.

**Methods:**

Between 1997 and 2017, patients in the Swedish region of Norrbotten with a diagnosis of bipolar or schizoaffective disorder were screened for all episodes of overt hyperthyroidism in form of thyrotoxicosis or thyroiditis. Incidence rates of episodes of hyperthyroidism per 1000 person-years (PY) were compared in relation to lithium exposure; concurrent, previous, or no exposure ever (lithium-naïve patients)

**Results:**

In 1562 patients, we identified 16 episodes of hyperthyroidism corresponding to an incidence rate of 0.88 episodes per 1000 PY. Ninety-four percent of episodes had occurred in women. Patients who had concurrently been exposed to lithium, had an incidence rate of 1.35 episodes per 1000 PY. Patients who had previously been exposed to lithium had an incidence rate of 0.79 per 1000 PY. Patients who had never been exposed to lithium had an incidence rate of 0.47 per 1000 PY. There were no significant differences in the risk ratios for patients with concurrent or previous exposure compared with lithium-naïve patients, neither for hyperthyroidism overall, thyrotoxicosis, or thyroiditis.

**Conclusion:**

Lithium-associated hyperthyroidism seems uncommon. The risk of hyperthyroidism does not seem significantly higher in patients with current or previous lithium exposure than in lithium-naïve patients.

## Introduction

Ever since the introduction of lithium as a treatment for bipolar disorders (BD), adverse effects of thyroid function have been a concern.^1,2^ Most commonly, lithium is associated with hypothyroidism. Prevalence estimates range from 14% to 17% for overt and from 19% to 35% for subclinical hypothyroidism.^3–5^ Lithium-associated hyperthyroidism is much rarer than lithium-associated hypothyroidism.^6,7^ Yet, it may be of substantial clinical significance for affected individuals. For instance, lithium-associated hyperthyroidism could destabilise mood, mimic manic episodes and impact physical health.^8^ At the same time, the underlying mechanism of action by which lithium could induce hyperthyroidism remains unclear. Indeed, lithium, due to its association with hypothyroidism, has been tried as a treatment for hyperthyroid states.^9,10^ The starting point for evaluating the clinical relevance of lithium-associated hyperthyroidism is understanding the magnitude of the problem. However, only few studies have explored the incidence of lithium-associated hyperthyroidism.^1,11–13^ Even fewer studies have explored the incidence of lithium-associated hyperthyroidism in comparison to unexposed individuals with or without BD.^6,11,13,14^ We found only one study that compared the incidence of lithium-associated hyperthyroidism with a population-based incidence.^15^ We did not find any study exploring hyperthyroidism in relation to previous lithium exposure. To avoid overestimation of the incidence, it is also important to establish the aetiology. Hyperthyroidism associated with lithium does not necessarily equate hyperthyroidism attributable to lithium. In other words, association does not necessarily imply causation since there may be other confounders or effect-modifying factors at play.

### Aims of this study

The aims of this study were to determine the impact of lithium treatment on the incidence of hyperthyroidism in patients with bipolar or schizoaffective disorder and assess its aetiology.

### Procedure

#### Study design

This study is part of LiSIE (Lithium – Study into Effects and Side Effects), a retrospective cohort study. LiSIE aims at identifying the best long-term treatment options for patients with bipolar and related conditions. Based on longitudinal medical records review, LiSIE explores the effects and potential adverse effects of lithium compared with other mood stabilisers. LiSIE was carried out according to the Declaration of Helsinki guidelines and was approved by the Regional Ethics Review Board at Umeå University, Sweden (DNR 2010-227-31M, DNR 2011-228-32M, DNR 2014-10-32M and DNR 2018-76-32M). The current work is the second in series within LiSIE exploring the relationship between hyperthyroid states and lithium. Whereas the first study examined the relationship between hyperthyroxinaemia, that is, increased free thyroxine (fT4) and lithium intoxication,^16^ the current work examines the relationship between hyperthyroidism and history of lithium exposure. We have described the method in detail in the first study.^16^ To ease understanding, we described the method in detail even here. We have summarised the whole method in a STROBE checklist (Appendix 1).

#### Sample

LiSIE invited all individuals in the Swedish regions of Västerbotten and Norrbotten ⩾ 18 years of age, who, according to the 10th revision of the International Statistical Classification of Diseases and Related Health Problems (ICD 10),^17^ had received a diagnosis of bipolar disorder (BD) (ICD10 F31) or schizoaffective disorder (SZD) (ICD10 F25), *or* who had used lithium as a mood stabiliser between 1997 and 2011.^18^ All participants were informed about the nature of the study in writing and provided verbal informed consent. The consent was documented in our research files, dated and signed by the research worker who obtained the consent. In accordance with the ethics approval granted, deceased patients were also included. The consent procedures were concluded at the end of 2012. The cohort was locked at this point; no new patients were included in the study thereafter.

#### Patient selection and inclusion criteria

**Figure 1.**
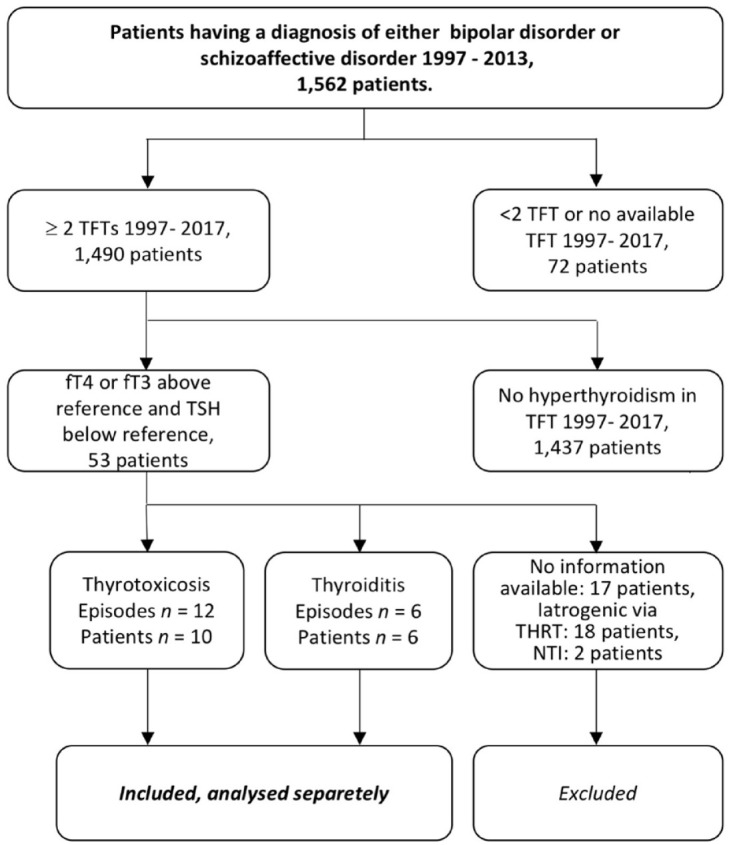
Selection of outcome. fT3, free triiodothyronine; fT4, free thyroxine; NTI, nonthyroid illness; TFT, Thyroid function test; THRT, thyroid hormone replacement therapy; TSH, thyroid stimulating hormone.

This study considered patients from the region of Norrbotten who had received a diagnosis of either BD or SZD. The diagnoses BD or SZD were assigned when a patient had received a diagnosis of either condition on at least two occasions at least 180 days apart. In line with ICD-10 classification, we also assigned a BD diagnosis when patients had experienced at least one manic and one depressive episode. To create subcategories of BD and SZD, the diagnoses of patients were validated further according to what they would have looked like in *DSM*-5. This has been described in detail in previous work on the LiSIE cohort.^19^ Four categories were considered, type-1 bipolar disorder (BD-I) (296.4), SZD (295.7), type-2 bipolar disorder (BD-II) (296.80) and other BD (296.89). This validation process for psychiatric diagnoses for the whole LiSIE cohort used clinical data up to 2015.^19,20^ We then determined all episodes of overt hyperthyroidism for the whole sample until 31 December 2017, the endpoint of the study (Figure 1). For all identified episodes of hyperthyroidism, we explored whether there were signs for mixed states or rapid cycling disorder according to *DSM*-5 until the endpoint in 2017. We also examined whether the symptoms pattern changed after the detection and treatment of hyperthyroidism, which could have warranted a reclassification of diagnosis.

#### Exclusion criteria

For the whole LiSIE study, we excluded patients in whom, after manual validation from the medical records, a diagnosis of schizophrenia or personality disorder was more likely than BD or SZD.

#### Outcome

The primary outcome was the number of episodes of overt hyperthyroidism. We expressed this as an incidence rate, that is, episodes per 1000 person-years (PY), depending on lithium exposure status. In the judgement of which episodes to include as clinically relevant ‘true’ episodes of overt hyperthyroidism, we used thyroid function tests (TFTs) as a starting point. TFTs were then put into context of the clinical assessment documented in the medical records.

As a necessary criterion for hyperthyroidism, a patient had to experience a decrease in thyroid stimulating hormone (TSH) *and* an increase in free thyroxine (fT4) or free triiodothyronine (fT3), consecutively at least twice within 6 months of each other. We also considered hyperthyroidism to be present when a patient had experienced a decrease in TSH and an increase in fT4 or fT3 on one occasion and had thereafter been started on treatment, leading to normalisation of TSH and fT4 or fT3 in the next test. Most TFTs were analysed with a Roche Diagnostics Scandinavia immunoassay with normal range reference values for thyroid function tests of 0.27–4.20 mIU/l for TSH, 12.0–22.0 pmol/l for fT4 and 3.5–6.5 pmol/l for fT3.

As a sufficient criterion for hyperthyroidism, the diagnosis of hyperthyroidism had to be endorsed in the clinical notes as (a) thyroiditis, a transient hyperthyroid state that normalised within weeks to 6 months at follow-up, or (b) thyrotoxicosis, a permanent auto-immune mediated hyperthyroid state that required treatment. For thyroiditis, we counted each episode. For thyrotoxicosis, we only counted the first episode, but not subsequent episodes. Subsequent episodes were considered relapses of the same condition only. We excluded episodes for which (a) there were less than two TFTs available, that is, either only TSH without matching fT4, or TSH and fT4 on only one occasion, or (b) TFTs could not be interpreted due to the lack of other clinical information, (c) thyroid hormone replacement therapy (THRT) had resulted in unintended (iatrogenic) hyperthyroidism, (d) hyperthyroidism had been a result of an overdose with thyroid hormones, or (e) hyperthyroidism had been transient as a result of nonthyroid illness. (Panel 1).

**Panel 1.** Events counted and not counted as overt hyperthyroidism.

Aetiology of overt hyperthyroidism	Counted as event	Description
Thyrotoxicosis	Yes	Referred to endocrinologists. TRAb positive. Iodine uptake scan positive.
Thyroiditis	Yes	Referred to endocrinologists, TRAb negative. No iodine uptake scan or negative result. Transient.
Iatrogenic hyperthyroidism	No	THRT induced. Reversible on lowered dose of THRT, transient, no other intervention / investigation.
Nonthyroid illness	No	Abnormal thyroid function test related to acute illness, as explicitly stated in medical records. Transient without intervention/investigation in relation to thyroid function.
Unknown	No	Hyperthyroidism in laboratory values, but thyroid status not commented upon in medical journal. As such of unclear aetiology and no follow-up.

THRT, thyroid hormone replacement therapy; TRAb, TSH-receptor antibody.

#### Time in the study in which the outcome was obtained

The outcome hyperthyroidism was determined over a 21-year period. Time in the study was measured in years from 1^st^ of January 1997 to 31 December 2017. In this time frame, for each patient, the observation time started at the time of diagnosis of BD/SZD, or at the time of continuous mood-stabiliser treatment. Continuous mood-stabiliser treatment was defined as exposure to three or more months of treatment with lithium, valproate, carbamazepine, lamotrigine, risperidone, aripiprazole, olanzapine ⩾ 7.5 mg per day, or quetiapine ⩾ 100 mg per day. For olanzapine and quetiapine, we used dose thresholds because these agents are often used nonspecifically at lower doses or on a when needed basis.^21^ In accordance with the set-up of the study we did not consider treatment times before the age of 18 years. For patients who died before 31 December 2017, the observation time stopped at the date of their death.

#### Exposure parameters

The main exposure parameter was lithium treatment. Proof of lithium exposure was determined by a lithium prescription on at least one occasion over 14 days and at least one blood lithium concentration of at least 0.2 mmol/l. We did not require lithium concentrations to be therapeutic because our objective was to determine an adverse effect of lithium treatment and not therapeutic effectiveness. For the same reason, we counted patients even if they had only received one prescription for lithium. Prior exposure to lithium was traced back in archived medical records until 1965.

For baseline characteristics, we stratified patients into four exposure groups. Group 1 concerned patients who had continuous lithium treatment during the study. Group 2 involved patients who had intermittently been exposed to lithium at some point during the study but had not taken lithium continuously. Group 3 included patients who had been exposed to lithium before but not after study start. Group 4 included patients who had never been exposed to lithium. For our definition of continuous treatment, we considered the first 3 months after discontinuation of lithium to be part of lithium exposure because lithium could still impact thyroid function.^18,22^

**Figure 2.**
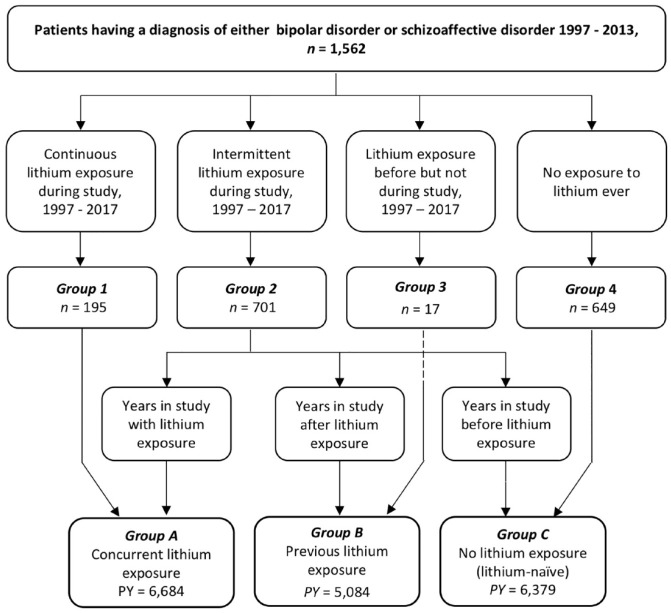
Counting of person-years. PY, person-years.

However, these groups could not be used for the calculation of incidence rates. The incidence rates depended on the PY of lithium exposure and patients in group 2 could move through different exposure states. Therefore, for the incidence rates, we created three groups according to the time spent in each lithium exposure state. Group A concerned PY accumulated while exposed to lithium, that is, concurrent lithium use. Group A included all PY from group 1 patients and PY from group 2 patients while lithium exposed. Group B included PY not currently but previously exposed to lithium. Group B included PY from group 2 after lithium discontinuation and all PY from group 3 patients. Group C included all PY without any lithium exposure ever, that is, lithium-naïve. Group C included PY of group 2 patients before the first exposure to lithium and all PY of group 4 patients (Figure 2).

#### Other variables

We also recorded age, sex, TSH-receptor antibodies (TRAb) and type of underlying mood disorder.

#### Chart review and validation

For the outcomes and exposure variables, we retrospectively reviewed the medical records of all eligible patients from 1997 to December 31, 2017. From the medical records, we manually validated the date of the electronic prescriptions when lithium or THRT had been started or discontinued.

#### Control for bias and missing data

We controlled for selection bias in the entire retrospective cohort study (LiSIE) using key parameters available in anonymized form. These included age, sex and, where applicable, maximum recorded concentrations of lithium and creatinine. In accordance with the ethics approval granted, we compared these parameters for consenting and nonconsenting patients. No significant differences were found between the two groups. The data was complete for included patients for the defined outcome.

#### Statistical analysis

Before analysis, the data were anonymized. Then, the data were analysed descriptively, giving medians for continuous variables and frequencies for categorical variables of the baseline characteristics. To analyse the relations between categorical variables, chi-square or Fisher’s exact tests were used. Differences in continuous variables with respect to groups were analysed with the Mann–Whitney U test.

We then compared episodes of hyperthyroidism with concurrent lithium exposure (group A) and episodes after previous lithium exposure (group B) with episodes with no lithium exposure, lithium-naïve (group C). The incidences in episodes per 1000 PY were calculated. Risk ratios were also calculated using group C as the basis for comparisons. The data was handled with SPSS version 27.0 (IBM, Armonk, NY, USA) and the analysis was conducted with MedCalc Software Ltd (Version 20.116).^23^ The significance level was set at a p value of 0.05 throughout.

## Results

### Baseline characteristics

1562 patients (62% women) were included in the study. Of these, 896 patients (60.2% women) had been exposed to lithium at any time during the review between 1997 and 2017. The groups 1 to 3 did not differ significantly in diagnosis compared with group 4 (no lithium exposure ever) as baseline. They differed significantly in age, depending on how long patients had been in the study. In the group with continuous lithium treatment, there were significantly more men than in group 4 (Table 1).

**Table 1.** Baseline characteristics.

	Whole group	Group 1 Continuous lithium exposure	Group 2 Intermittent lithium exposure	Group 3 Lithium exposure before but not during study	Group 4 No lithium exposure ever
Patients (*n*)	1562	195	701	17	649
Age at start of study					
Median, (min–max)	42.0 (18–92)	48.0 (18–92)*	43.0 (18–84)**	55.0 (25–79)*	39.0 (18–91)
Sex (%)					
Women	62.1	54.9***	61.6	70.6	64.6
Men	37.9	45.1***	38.4	29.4	35.4
Diagnosis (%)					
BD-I	12.3	21.0*	14.1*	23.5***	7.2
SZD	14.8	17.9	12.6	11.8	15.4
BD-II	52.2	36.9*	53.9	35.3	55.2
BD other	20.7	24.1	18.4	29.4	22.2

BD, bipolar disorder; SZD, schizoaffective disorder.

* *p* < 0.001, **p ⩽ 0.01, ***p = < 0.05, groups 1 to 3 against group 4 as the baseline.

### Episodes of hyperthyroidism

In total, there were 16 episodes of hyperthyroidism in 16 patients, the characteristics of which are summarised in Table 2. Of all episodes, 93.7% occurred in women. In 12.5% of episodes, there was a diagnosis of SZD; in none of the episodes, there was a diagnosis of BD-I. For none of the episodes, rapid cycling or mixed states were recorded. After onset of hyperthyroidism, one patient changed from symptoms suggestive of BD-II to symptoms suggestive of attention deficit/hyperactivity disorder (ADHD).

**Table 2.** Patients with episodes of hyperthyroidism.

Patients (*n*)	16
Age at start of study	
Median (min–max)	46.5 (24–70)
Age at onset of hyperthyroidism	
Median (min–max)	54.5 (28–83)
Sex (%)	
Women	93.7
Men	6.3
Diagnosis (%)^a^	
BD-I	0
SZD	12.5
BD-II	62.5
BD-other	25.0
Mixed states (%)^b^	0
Rapid cycling (%)^b^	0
Type of hyperthyroidism, *n* (%)^b^	
Thyrotoxicosis	62.5
Thyroiditis	37.5
Change in symptom pattern after the detection and treatment of hyperthyroidism (%)^b^	6.3
Previous episodes of hypothyroidism resulting in hormone replacement therapy (%)^b^	12.5

BD, bipolar disorders; SZD, schizoaffective disorder.

a Up to 31 December 2015.

b Up to 31 December 2017.

#### Person-years

Table 3 lists the PY allocated to each group with median time in the study.

**Table 3.** Person-years spent in groups.

	Person-years		
	Group A Concurrent lithium exposure	Group B Previous lithium exposure	Group C Lithium-naïve (Baseline)
Group 1 Continuous lithium exposure	1463	–	–
Group 2 Intermittent lithium exposure	5221	4968	422
Group 3 Lithium exposure before but not during study	–	116	–
Group 4 Never exposed to lithium	–	–	5957
Total person-years	6684	5084	6379
Time in study, years			
Median (min–max)	5.7 (0–21)	6.2 (0–21)	8.8 (0–21)

For the whole sample, there were 18,147 observed PY. This yielded an incidence rate of 0.88 episodes of hyperthyroidism per 1000 PY. Of the 16 episodes of hyperthyroidism, six (37.5%) concerned thyroiditis. Excluding these, the incidence rate for thyrotoxicosis was 0.55 per 1000 PY. In group A with concurrent lithium exposure, there were nine episodes of hyperthyroidism in nine patients. The incidence rate was 1.35 episodes per 1000 PY. In group B with previous lithium exposure, there were four episodes of hyperthyroidism. This yielded an incidence rate of 0.79 per 1000 PY. In the lithium-naïve group C, there were three patients with three episodes of hyperthyroidism. The incidence rate was 0.47 episodes per 1000 PY (Table 4). There were no significant differences in the risk ratios in groups A or B with concurrent or prior lithium exposure compared with the lithium-naïve group C.

**Table 4.** Incidence rates of hyperthyroidism according to lithium treatment status.

	Group A Concurrent lithium exposure	Group B Previous lithium exposure	Group C Lithium-naïve (Baseline)
PY	6684	5084	6379
Hyperthyroidism (thyrotoxicosis + thyroiditis)			
Episodes (*n*)	9	4	3
Incidence rate per 1000 PY	1.35	0.79	0.47
RR (CI) *p*-value	2.86 (0.72–16.44) 0.108	1.67 (0.28–11.42) 0.523	–
ARR (CI) *p*-value	0.88 (−0.16–1.92) 0.099	0.32 (−0.59–1.37) 0.496	–
Sex Women [*n* (%)]	8 (88.9)	4 (100.0)	3 (100.0)
Age at hyperthyroidism [Median (min–max)]	62.0 (29–83)	48.0 (39–60)	38.0 (28–51)
Thyrotoxicosis only			
Episodes (*n*)	5	3	2
Incidence rate per 1000 PY	0.75	0.59	0.31
RR (CI) *p*-value	2.39 (0.39–25.06) 0.317	1.88 (0.22–22.53) 0.520	–
ARR (CI) *p*-value	0.43 (−0.36–1.23) 0.284	0.28 (−0.49–1.05) 0.481	–
Thyroiditis only			
Episodes (*n*)	4	1	1
Incidence rate per 1000 PY	0.60	0.20	0.16
RR (CI) *p*-value	3.82 (0.38–188.00) 0.238	1.25 (0.02–98.49) 0.887	–
ARR (CI) *p*-value	0.44 (−0.23–1.11) 0.197	0.04 (−0.05–0.05) 0. 872	–

ARR, absolute risk reduction with group C as the baseline; CI, 95% confidence interval; PY, person-years; RR, risk ratio with group C as the baseline.

## Discussion

### Findings

In our study, we compared incidence rates of confirmed hyperthyroidism between groups based on lithium exposure. To our knowledge, this is the first study in which the incidence rate of hyperthyroidism is not only established for patients with concurrent lithium exposure, but also for patients with previous lithium exposure. We found an incidence rate of hyperthyroidism (thyrotoxicosis and thyroiditis) of 1.35 per 1000 PY in patients with concurrent lithium exposure. However, this incidence rate was not significantly higher than for patients with previous exposure to lithium or lithium-naïve patients. Neither did lithium exposure status significantly affect the incidence rates for thyrotoxicosis or thyroiditis as separate diagnostic entities. A previous population-based study in our catchment area found an incidence of thyrotoxicosis in the catchment area of our study of 0.47 per 1000 inhabitants per year.^24^ The estimate in that study is based on number of individuals, that is, incidence risk, whereas the estimates in our study are based on number of PY, that is, incidence rate. Taking the methodological difference into account the estimates seem comparable in magnitude.

#### Comparison with occurrence of hypothyroidism in the LiSIE cohort

In previous work, we determined the occurrence of hypothyroidism in the LiSIE cohort.^25^ In the same 21-year review period, 18.0% of patients received THRT after the diagnosis of BD/SZD or after mood stabiliser start. Here, we excluded patients who received thyroid hormones for augmentation treatment of depression or in the context of pregnancy. In patients exposed to lithium, 23.9% received THRT for either overt or subclinical hypothyroidism. This was in line with ranges given by previous studies.^26–28^

As could be expected, in our cohort, hypothyroidism was much more common than hyperthyroidism. Of the 16 cases of hyperthyroidism meeting our inclusion criteria, two patients had been diagnosed and treated for hypothyroidism previously. In both cases, hyperthyroidism was not related to THRT.

#### Comparison with other studies of lithium-associated hyperthyroidism

In previous studies, estimates of incidence rates of lithium-associated hyperthyroidism range from 0.08 to 7.8 per 1000 PY.^1,11–13^ Part of this variation could be due to different definitions applied. In some studies, hyperthyroidism was defined through the requirement of anti-thyroid treatment.^1,13^ Other studies relied on a recorded diagnosis of hyperthyroidism o*r* a TSH < 0.1 mU/l,^11^ clinical characteristics of thyrotoxicosis and abnormal biochemical findings *or* nodular goitre associated with lithium therapy,^12^ radioiodine uptake,^15^ or a TSH < 0.2 mU/l.^6^ A further study based on radioiodine uptake scans found an incidence rate of lithium-associated thyrotoxicosis of 2.7 cases per 1000 PY and of lithium-associated thyroiditis of 1.3 per 1000 PY.^15^ In some previous studies, lithium-associated hyperthyroidism had been reported more commonly in women.^13^ This, we also saw in our study. In our study, the proportion of men continuously treated with lithium was higher than the proportion of men never been treated with lithium. It is possible that men without lithium had received fewer TFTs since thyroid dysfunction is more common in women. But since there were only few cases of hyperthyroidism in absolute terms, it is unlikely that incidence rates would have changed substantially. Finally, risk estimates for lithium associated hyperthyroidism may also be inflated. Individuals treated with lithium are much more likely to receive regular testing of the thyroid function than a nonexposed population. This way, many more transient hyperthyroid states (thyroiditis) will be picked up that would otherwise have gone undetected.

#### Aetiology and clinical relevance of lithium-associated hyperthyroidism

According to the Council for International Organisations for Medicinal Sciences (CIOMS) grading,^29^ the incidence rate of hyperthyroidism in patients exposed to lithium constituted an uncommon or infrequent event in our study. The incidence rates of hyperthyroidism in patients previously or never exposed to lithium constituted rare events. Ultimately, when comparing risk ratios in our cohort, lithium exposure did not significantly increase the risk of hyperthyroidism, neither in relative terms (risk ratio), nor in absolute terms (absolute risk reduction). Therefore, we could not refute the assumption that any differences in incidence rates might be due to chance alone.

Indeed, the mechanism by which lithium could induce a hyperthyroid state remains unclear.^8^ Several mechanisms have been suggested, including (a) an autoimmune process,^30^ (b) iodine retention leading to an expansion of intrathyroidal iodine stores,^12^ or (c) a direct toxic effect on the thyroid gland.^15,31^ In our study, previous lithium exposure did not lead to a significant increase of hyperthyroidism risk. Based on our findings, it is unlikely that previous lithium exposure has triggered an autoimmune process that then took its course even after lithium was discontinued. Equally unlikely is a substantial toxic effect. If toxicity had led to immediate damage, this should have been visible in patients concurrently exposed. If toxicity had led to a delayed damage, this should have been visible in patients previously exposed. Iodine retention as a potential mechanism of hyperthyroidism has been suggested but remains speculative.^12^

Conversely, lithium has been tested as treatment of hyperthyroidism as potential inhibitor of thyroid hormone release.^32^ Several small studies have compared the effects of radioiodine treatment monotherapy with radioiodine and lithium in combination in patients with thyrotoxicosis. A meta-analysis of nine such trials with a total of 928 patients with mostly Grave’s disease did not show any significant difference regarding these two treatment options. In subgroup analysis, higher cumulative doses of 5000 to 6500mg lithium carbonate over 7 days showed a significantly improved curation rate in term of recovery to a euthyroid or hypothyroid state.^33^

#### Comparison with studies of bipolar disorder-associated hyperthyroidism

The mechanisms by which hyperthyroid states and BD could interact also remain unclear. It has been suggested that affective disorders in general and BD in particular are more common in individuals with hyperthyroidism.^34,35^ The mechanism could be an inflammatory process following hyperthyroidism, which would then induce BD. Alternatively, a residual disturbance in mood following an episode of hyperthyroidism could predispose individuals to develop BD.^34^ Although BD is uncommon in thyroid dysfunction,^36^ twin studies have found that autoimmune thyroiditis may be associated with a genetic vulnerability for BD, but not to BD itself.^37^

Some studies suggest there might be more circulating antibodies targeting thyroid tissue in individuals with BD.^5^ This could then lead to an increased risk of rapid cycling.^38,39^ Yet, hyperthyroidism in itself has not convincingly been associated rapid cycling^39^ or manic episodes.^40,41^ Furthermore, no significant differences have been found in individuals with BD with and without abnormal thyroid function regarding various mood states, neither mixed features,^42,43^ nor euthymic, depressed, or manic states.^43^ Ultimately, the association between autoimmune thyroid dysfunction with BD remains unclear.^37^

In any event, abnormal thyroid function tests are common and can occur in up to one in every three psychiatric patients without reflecting actual thyroid disease.^44^ This again may be due to more frequent testing of thyroid function and identifying more cases of silent and/or silent or transient, thyroiditis,^15^ thereby inflating incidence rates for patients with BD. Finally, even if a potential association between BD and hyperthyroidism was established in future, this would weaken but not strengthen the assumption of an association between lithium and hyperthyroidism.

#### Background incidence of thyrotoxicosis reported in other countries

Incidence rates of thyrotoxicosis in the general population in other countries, have been estimated to lie between 0.3 and 0.8 cases per 1000 PY.^45,46^ The incidence of silent thyroiditis has been estimated to range from less than 0.03 to 0.28 cases per 1000 PY.^15^ Our incidence rate for thyroiditis patients with BD or SZD in the lithium-naïve group was within this range (0.16 per 1000 PY). Based on incidence figures alone, an association between BD and hyperthyroidism seems unlikely or very weak, limiting its relevance. A study from Taiwan found an incidence rate of BD of 1.6 per 1000 PY in patients with hyperthyroidism and of 0.7 per 1000 PY in patients without hyperthyroidism. This constituted a significantly risk of BD in patients with hyperthyroidism with an incidence rate ratio of 2.31 (95% CI 1.80–2.99).^34^ But this study did not give any indication of which came first, BD or hyperthyroidism. Neither did this study distinguish between lithium and other mood stabilisers.

### Strengths

For the whole LiSIE study consent was high so that we could include 82% of eligible patients. For this particular study, we had available up to 21 years of real-life validated data for 1562 patients. Access to laboratory data, prescription data and medical records made it possible to establish the exact time of exposure to lithium so that we could calculate incidence rates in PY. From the available data sources, we could also map the chronology of episodes to determine what came first, hyperthyroidism or lithium treatment. Having access to detailed clinical data and not only to laboratory or prescription data made it possible to establish the aetiology and then stratify episodes of hyperthyroidism accordingly to assess clinical relevance.

### Weaknesses

The study was observational and retrospective in nature. This limited our ability to establish causality. However, due to the rarity of the outcome, the study did not yield itself to a prospective design or randomised controlled trial. Relying on retrospective analysis of recorded clinical information, the quality of the study was limited by the quality of information recorded. For instance, time spent in manic or depressed episodes required to assess predominant polarity would be difficult to reliably derive from retrospective medical records. However, we could check for symptom changes after onset of hyperthyroidism.

There were TFT indicating possible hyperthyroidism with no available information or follow-up. These episodes were excluded. We could therefore potentially have missed cases of thyroiditis in cases with transient TFT with no intervention or follow-up in the following 6 months. However, it is unlikely that we would have missed cases of thyrotoxicosis. If we had counted these excluded cases, the CIOMS categories would have remained unchanged, that is, uncommon (1.80 per 1000 PY) for concurrent lithium treated patients. The CIOMS categories would have changed from rare to uncommon for lithium-naïve patients (2.16 per 1000 PY) and patients previously been treated with lithium (1.57 per 1000 PY). The risk ratios for incidence-rates (concurrent or previous lithium exposure *versus* lithium-naïve) would have remained nonsignificant (RR 1.15, 95% CI 0.45–2.96, *p* = 0.756, and RR 1.38, 95% CI 0.53–3.63, *p* = 0.468). Finally, in an ideal world, we would have controlled for potential confounders. However, as there were only very few (*n* = 16) episodes of overt hyperthyroidism, a more in-depth analysis, controlling for various potential confounders, would have resulted in overfitting.

## Conclusion

Our study suggests that lithium-associated hyperthyroidism in patients with BD or SZD is uncommon, and that the risk of hyperthyroidism does not seem to be higher than in lithium-naïve patients. Still, occasional cases of thyrotoxicosis and thyroiditis can occur, but not more likely than in the general population. Therefore, clinicians should try to determine the underlying aetiology of hyperthyroid states with an open mind instead of automatically attributing hyperthyroidism to lithium treatment. As hyperthyroidism is treatable in the case of thyrotoxicosis, and transient or reversible with treatment in the case of thyroiditis, clinicians should not withhold lithium for fear of hyperthyroidism as an adverse effect. Closer monitoring for hyperthyroidism is unlikely to improve clinical outcomes but may conversely increase the risk overtreatment by picking up more transient states.

## Supplemental Material

sj-docx-1-tpp-10.1177_20451253231151514 – Supplemental material for Incidence of hyperthyroidism in patients with bipolar or schizoaffective disorder with or without lithium: 21-year follow-up from the LiSIE retrospective cohort study Click here for additional data file. Supplemental material, sj-docx-1-tpp-10.1177_20451253231151514 for Incidence of hyperthyroidism in patients with bipolar or schizoaffective disorder with or without lithium: 21-year follow-up from the LiSIE retrospective cohort study by Ingrid Lieber, Michael Ott, Robert Lundqvist, Mats Eliasson and Ursula Werneke in Therapeutic Advances in Psychopharmacology

## Declarations

**Ethics approval and consent to participate:** The LiSIE study was conducted according to the guidelines of the Declaration of Helsinki and was approved by the Regional Ethics Review Board at Umeå University, Sweden (DNR 2010-227-31M, DNR 2011-228-32M, DNR 2014-10-32M, DNR 2018-76-32M). Informed consent was obtained from all subjects in the study. In accordance with the ethics approval granted, no consent was obtained for deceased patients.

**Consent for publication:** Not applicable. This work was the second in series within LiSIE exploring the relationship between hyperthyroid states and lithium. We have described the method in detail in the first study (Lieber 2022,^16^ manuscript ID JCM11113041), which has been published as an open access article distributed under the terms and conditions of the Creative Commons Attribution (CC BY) licence (https://creativecommons.org/licenses/by/4.0/). To ease understanding, we described the method in detail even in the current work.

**Availability of data and materials:** The datasets generated and/or analysed during the current study are not publicly available due to lack of ethics committee permission and not having been part of the consent process. The structure of the dataset and the coding specification are available from the authors. Any other reasonable request will be raised with the healthcare provider.
