# Supplementary material for: Incidence of hyperthyroidism in patients with bipolar or schizoaffective disorder with or without lithium: 21-year follow-up from the LiSIE retrospective cohort study
Source: Ther Adv Psychopharmacol. 2023 Feb 9;13:20451253231151514. doi: 10.1177/20451253231151514 (PMC9912559; doi:10.1177/20451253231151514)
Supplement: sj-docx-1-tpp-10.1177_20451253231151514 – Supplemental material for Incidence of hyperthyroidism in patients with bipolar or schizoaffective disorder with or without lithium: 21-year follow-up from the LiSIE retrospective cohort study [file sj-docx-1-tpp-10.1177_20451253231151514.docx]

**Appendix 1: STROBE Statement - checklist for our study**

| **STROBE requirement** | **#** | **Our study** |
| --- | --- | --- |
| *Title and abstract* | 1 |  |
| *(a)* Indicate the study’s design with a commonly used term in the title and abstract |  | 1. Given: Title: “Incidence of hyperthyroidism in patients with bipolar or schizoaffective disorder with or without lithium – 21-year follow-up from the LiSIE retrospective cohort study”,   Abstract: “Design: This study is part of the LiSIE (Lithium – Study into Effects and Side Effects) retrospective cohort study.” |
| *(b)* Provide in the abstract an informative and balanced summary of what was done and what was found |  | (b) Structured abstract is provided. |
| *Introduction* |  |  |
| Background/rationale: Explain the scientific background and rationale for the investigations being reported | 2 | Background is outlined in introduction. |
| Objectives:  State specific objectives, including any pre-specified hypotheses | 3 | Aims stated in the text, “The aims of this study were to determine the impact of lithium treatment on the incidence of hyperthyroidism in patients with bipolar or schizoaffective disorder and assess its aetiology.” |
| *Methods* |  |  |
| Study design:  Present key elements of the study design early in the paper | 4 | Study design: the study is part of LiSIE (Lithium – Study into Effects and Side Effects), a retrospective cohort study aimed at identifying the best long-term treatment options for patients with bipolar and related conditions. Key elements of the study included in the manuscript: study design, participants, selection: inclusion and exclusion criteria, outcome definition, exposure parameters, variable definitions, validation process, control for bias, missing data and statistical analysis. |
| Setting:  Describe the setting, locations, and relevant dates, including periods of recruitment, exposure, follow-up, and data collection | 5 | Setting and all relevant dates described in manuscript: “LiSIE invited all individuals in the Swedish regions of Västerbotten and Norrbotten ≥18 years of age, who, according to the 10th revision of the International Statistical Classification of Diseases and Related Health Problems (ICD 10), had received a diagnosis of bipolar disorder (BD) (ICD10 F31) or schizoaffective disorder (SZD) (ICD10 F25), or who had used lithium as a mood stabiliser between 1997 and 2011.”  “The current study considered patients from the region of Norrbotten who had received a diagnosis of either BD or SZD. The diagnoses BD or SZD were assigned when a patient had received a diagnosis of either condition on at least two occasions at least 180 days apart. In line with ICD-10 classification, we also assigned a BD diagnosis when patients had experienced at least one manic and one depressive episode. To create subcategories of BD and SZD, the diagnoses of patients were validated further according to what they would have looked like in DSM-5. This has been described in detail in previous work on the LiSIE cohort (19). Four categories were considered, type-1 bipolar disorder (BD-I) (296.4), SZD (295.7), type-2 bipolar disorder (BD-II) (296.80) and other BD (296.89). This validation process for psychiatric diagnoses for the whole LiSIE cohort used clinical data up to 2015 (19, 20). We then determined all episodes of overt hyperthyroidism for the whole sample until 31 December 2017, the endpoint of the study.” |
| Participants:  *(a)* Give the eligibility criteria, and the sources and methods of case ascertainment and control selection. Give the rationale for the choice of cases and controls  (*b*) For matched studies, give matching criteria and the number of controls per case | 6 | (a) As above  “For the outcomes and exposure variables, we retrospectively reviewed the medical records of all eligible patients from 1997 to December 31, 2017. From the medical records, we manually validated the date of the electronic prescriptions when lithium or thyroid hormone replacement therapy had been started or discontinued.    (b) N/A. |
| Variables:  Clearly define all outcomes, exposures, predictors, potential confounders, and effect modifiers. Give diagnostic criteria, if applicable | 7 | Definition for exposures and variables given in text. “T The primary outcome was the number of episodes of overt hyperthyroidism. We expressed this as an incidence rate, i.e., episodes per 1,000 person-years (PY), depending on lithium exposure status. In the judgement of which episodes to include as clinically relevant “true” episodes of overt hyperthyroidism, we used thyroid function tests (TFTs) as a starting point. TFTs were then put into context of the clinical assessment documented in the medical records..  As a necessary criterion for hyperthyroidism, a patient had to experience a decrease in thyroid stimulating hormone (TSH) and an increase in free thyroxine (fT4) or free triiodothyronine (fT3), consecutively at least twice within six months of each other. We also considered hyperthyroidism to be present when a patient had experienced a decrease in TSH and an increase in fT4 or fT3 on one occasion and had thereafter been started on treatment, leading to normalisation of TSH and fT4 or fT3 in the next test. Most TFTs were analysed with a Roche Diagnostics Scandinavia immunoassay with normal range reference values for thyroid function tests of 0.27 – 4.20 mIU/L for TSH, 12.0 – 22.0 pmol/L for fT4, and 3.5 – 6.5 pmol/L for fT3”.  Time in the study in which the outcome was obtained: “The outcome hyperthyroidism was determined over a 21-year period. Time in the study was measured in years from 1st of January 1997 to 31st of December 2017. In this time frame, for each patient, the observation time started at the time of diagnosis of BD/SZD, or at the time of continuous mood-stabiliser treatment. Continuous mood-stabiliser treatment was defined as exposure to three or more months of treatment with lithium, valproate, carbamazepine, lamotrigine, risperidone, aripiprazole, olanzapine ≥7.5 mg per day, or quetiapine ≥100 mg per day. For olanzapine and quetiapine, we used dose thresholds because these agents are often used non-specifically at lower doses or on a when needed basis (21). In accordance with the set-up of the study we did not consider treatment times before the age of 18 years. For patients who died before 31st of December 2017, the observation time stopped at the date of their death.  Exposure parameters: “The main exposure parameter was lithium treatment. Proof of lithium exposure was determined by a lithium prescription on at least one occasion over 14 days and at least one blood lithium concentration of at least 0.2 mmol/L. We did not require lithium concentrations to be therapeutic because our objective was to determine an adverse effect of lithium treatment and not therapeutic effectiveness. For the same reason, we counted patients even if they had only received one prescription for lithium. Prior exposure to lithium was traced back in archived medical records until 1965.”  Stratification based on exposure: “For baseline characteristics, we stratified patients into four exposure groups. Group 1 concerned patients who had continuous lithium treatment during the study. Group 2 involved patients who had intermittently been exposed to lithium at some point during the study but had not taken lithium continuously. Group 3 included patients who had been exposed to lithium before but not after study start. Group 4 included patients who had never been exposed to lithium. For our definition of continuous treatment, we considered the first three months after discontinuation of lithium to be part of lithium exposure because lithium could still impact thyroid function.    However, these groups could not be used for the calculation of incidence rates. The incidence rates depended on the PY of lithium exposure and patients in group 2 could move through different exposure states. Therefore, for the incidence rates, we created three groups according to the time spent in each lithium exposure state. Group A concerned PY accumulated whilst exposed to lithium, i.e. concurrent lithium use. Group A included all PY from group 1 patients and PY from group 2 patients whilst lithium exposed. Group B included PY not currently but previously exposed to lithium. Group B included PY from group 2 after lithium discontinuation and all PY from group 3 patients. Group C included all PY without any lithium exposure ever, i.e., lithium-naïve. Group C included PY of group 2 patients before the first exposure to lithium and all PY of group 4 patients “  We also recorded age and sex for episodes of hyperthyroidism. |
| Data sources  /measurement:  For each variable of interest, give sources of data and details of methods of assessment (measurement). Describe comparability of assessment methods if there is more than one group | 8 | Data source for all variables: electronic medical records.  Definition for each variable given in text. |
| Bias:  Describe any efforts to address potential sources of bias | 9 | Potential sources of bias discussed, including selection and observer bias.  “We controlled for selection bias in the entire retrospective cohort study (LiSIE) using key parameters available in anonymized form. These included age, sex, and, where applicable, maximum recorded concentrations of lithium and creatinine. In accordance with the ethics approval granted, we compared these parameters for consenting and non-consenting patients. No significant differences were found between the two groups”. |
| Study Size:  Explain how the study size was arrived at | 10 | Cf. figure 2, Result in ‘Baseline characteristics’ section:  “1,562 patients (62% women) were included in the study”. |
| Quantitative variables: Explain how quantitative variables were handled in the analyses. If applicable, describe which groupings were chosen and why | 11 | Main outcome: Outcome summarized in the following categories:  (1) Incidence of hyperthyroidism (thyrotoxicosis + thyroiditis) per 1,000 person-years,  (2) Incidence of thyrotoxicosis per 1,000 person-years,  (3) Incidence of thyroiditis per 1,000 person-years.  The results were presented in text and in Table 4., Results were grouped into 3 groups after lithium exposure; Group A (concurrent lithium exposure), Group B (previous lithium exposure) and Group C (lithium-naïve). |
| Statistical methods: *a)* Describe all statistical methods, including those used to control for confounding  *(b)* Describe any methods used to examine subgroups and interactions  (*c)* Explain how missing data were addressed  *(d)* If applicable, explain how matching of cases and controls was addressed  *(e)* Describe any sensitivity analyses | 12 | 1. Described in the ‘Statistical analysis’ section: “the data were analysed descriptively, giving medians for continuous variables and frequencies for categorical variables of the baseline characteristics. To analyse the relations between categorical variables, chi-square or Fisher’s exact tests were used. Differences in continuous variables with respect to groups were analysed with the Mann-Whitney U test. 2. We then compared episodes of hyperthyroidism with concurrent lithium exposure (group A) and episodes after previous lithium exposure (group B) with episodes with no lithium exposure, lithium-naïve (group C). The incidences in episodes per 1,000 PY were calculated. Risk ratios were also calculated using group C as the basis for comparisons. The data was handled with SPSS version 27.0 (IBM, Armonk, NY, USA) and the analysis was conducted with MedCalc Software Ltd (Version 20.116). The significance level was set at a p value of 0.05 throughout.” 3. See above. 4. N/A |
| *Results* |  |  |
| Participants:  *(a)* Report numbers of individuals at each stage of study—eg numbers potentially eligible, examined for eligibility, confirmed eligible, included in the study, completing follow-up, and analyzed  *(b)* Give reasons for non-participation at each stage  *(c)* Consider use of a flow diagram | 13 | (a+b) Of 1,562 included patients with BD or SZD were included for calculation of observed person-years. (Figure 2). Out of these 1,490 patients had thyroid function tests (TFTs) taken and were available for analysis of potential outcome.  (c) Flow diagram included in the manuscript as figure 1 and figure 2. |
| Descriptive data:  *(a)* Give characteristics of study participants (e.g. demographic, clinical, social) and information on exposures and potential confounders  *(b)* Indicate number of participants with missing data for each variable of interest | 14 | (a) Baseline characteristics described in table 1 of the manuscript.  (b) Included in the figure 1 and in the text. |
| Outcome data:  Report numbers in each exposure category, or summary measures of exposure | 15 | Outcome data presented in text and in table 4. |
| Main results  *(a)* Give unadjusted estimates and, if applicable, confounder-adjusted estimates and their precision (eg, 95% confidence interval). Make clear which confounders were adjusted for and why they were included  *(b)* Report category boundaries when continuous variables were categorized  *(c)* If relevant, consider translating estimates of relative risk into absolute risk for a meaningful time period | 16 | (a) Results presented according to the statistical method outlined in item 12  (b) Results presented according to the statistical method outlined in item 12. Variable definitions given in method.  (c) N/A |
| Other analysis:  Report other analyses done—e.g. analyses of subgroups and interactions, and sensitivity analyses | 17 | Sub-analysis according to lithium exposure, cf. item 12 |
| *Discussion* |  |  |
| Key results:  Summarize key results with reference to study objectives | 18 | Done |
| Limitations:  Discuss limitations of the study, taking into account sources of potential bias or imprecision. Discuss both direction and magnitude of any potential bias | 19 | Limitation discussed in regard to selection bias, data quality, and potential for observer bias/recording error. |
| Interpretation:  Give a cautious overall interpretation of results considering objectives, limitations, multiplicity of analyses, results from similar studies, and other relevant evidence | 20 | Results discussed in view of the limitations (weaknesses) of our study design. Advantages and disadvantages of studies based on medical records compared to register studies and definition of hyperthyroidism discussed. |
| Generalisability:  Discuss the generalizability (external validity) of the study results | 21 | Discussed in the context of bias. The sample for the study is judged to be representative. |
| Funding:  Give the source of funding and the role of the funders for the present study and, if applicable, for the original study on which the present article is based | 22 | This work was supported by a grant of the Research & Development Fund and VISARE NORR (Northern County Councils Regional Federation Fund) of Norrbotten Region, Sweden.  Conflict to interest statement for all authors included in manuscript. |

Source: <http://www.strobe-statement.org/>. Accessed 12 March 2019
